# A Genome-Wide Assessment of the Role of Untagged Copy Number Variants in Type 1 Diabetes

**DOI:** 10.1371/journal.pgen.1004367

**Published:** 2014-05-29

**Authors:** Manuela Zanda, Suna Onengut-Gumuscu, Neil Walker, Corina Shtir, Daniel Gallo, Chris Wallace, Deborah Smyth, John A. Todd, Matthew E. Hurles, Vincent Plagnol, Stephen S. Rich

**Affiliations:** 1 University College London (UCL) Genetics Institute (UGI), London, United Kingdom; 2 Wellcome Trust Sanger Institute, Hinxton, United Kingdom; 3 University of Virginia, Charlottesville, Virginia, United States of America; 4 JDRF/Wellcome Trust Diabetes and Inflammation laboratory, Cambridge Institute for Medical Research, University of Cambridge, Cambridge, United Kingdom; Yale School of Medicine, United States of America

## Abstract

Genome-wide association studies (GWAS) for type 1 diabetes (T1D) have successfully identified more than 40 independent T1D associated tagging single nucleotide polymorphisms (SNPs). However, owing to technical limitations of copy number variants (CNVs) genotyping assays, the assessment of the role of CNVs has been limited to the subset of these in high linkage disequilibrium with tag SNPs. The contribution of untagged CNVs, often multi-allelic and difficult to genotype using existing assays, to the heritability of T1D remains an open question. To investigate this issue, we designed a custom comparative genetic hybridization array (aCGH) specifically designed to assay untagged CNV loci identified from a variety of sources. To overcome the technical limitations of the case control design for this class of CNVs, we genotyped the Type 1 Diabetes Genetics Consortium (T1DGC) family resource (representing 3,903 transmissions from parents to affected offspring) and used an association testing strategy that does not necessitate obtaining discrete genotypes. Our design targeted 4,309 CNVs, of which 3,410 passed stringent quality control filters. As a positive control, the scan confirmed the known T1D association at the *INS* locus by direct typing of the 5′ variable number of tandem repeat (VNTR) locus. Our results clarify the fact that the disease association is indistinguishable from the two main polymorphic allele classes of the *INS* VNTR, class I-and class III. We also identified novel technical artifacts resulting into spurious associations at the somatically rearranging loci, T cell receptor, *TCRA/TCRD and TCRB*, and Immunoglobulin heavy chain, *IGH*, loci on chromosomes 14q11.2, 7q34 and 14q32.33, respectively. However, our data did not identify novel T1D loci. Our results do not support a major role of untagged CNVs in T1D heritability.

## Author Summary

For many complex traits, and in particular type 1 diabetes (T1D), the genome-wide association study (GWAS) design has been successful at detecting a large number of loci that contribute disease risk. However, in the case of T1D as well as almost all other traits, the sum of these loci does not fully explain the heritability estimated from familial studies. This observation raises the possibility that additional variants exist but have not yet been found because they have not effectively been targeted by the GWAS design. Here, we focus on a specific class of large deletions/duplications called copy number variants (CNVs), and more precisely to the subset of these loci that mutate rapidly, which are highly polymorphic. A consequence of this high level of polymorphism is that these variants have typically not been captured by previous GWAS studies. We use a family based design that is optimized to capture these previously untested variants. We then perform a genome-wide scan to assess their contribution to T1D. Our scan was technically successful but did not identify novel associations. This suggests that little was missed by the GWAS strategy, and that the remaining heritability of T1D is most likely driven by a large number of variants, either rare of common, but with a small individual contribution to disease risk.

## Introduction

Type 1 diabetes (T1D) is a complex common autoimmune disorder that is diagnosed in approximately 1 in 350 children in the UK [1], [2]. Its etiology arises from the action of multiple genetic and environmental risk factors [3], [4], with nearly one-half of the genetic risk residing in the human HLA-Major Histocompatibility Complex (MHC). In addition to the MHC, genome-wide association studies (GWAS) have identified over 40 loci robustly associated loci ([5], [6] and www.t1dbase.org). As for most complex human traits, T1D heritability and the extent of this heritability that remains to be discovered, are topics of debate [7]. While the gap between what is explained by known loci and true heritability is likely to be relatively small compared to other complex disorders [8], it is clear that additional variants remain to be found, with the potential to bring novel insights into T1D etiology. However, discovery of these variants will require either a significant sample size increase from previous well-powered GWASes [5], or alternative strategies that can target variants not captured by the standard GWAS design.

GWAS studies rely on a tag single nucleotide polymorphisms (SNPs) strategy to capture the extent of variation of the human genome and identify association signals. Variants poorly tagged by GWAS tag SNPs are therefore of primary interest for association purposes. Putative rare variants with high effect sizes fall into this category [9], [10]. Another class of variation that can be poorly tagged by the GWAS design are copy number variants (CNVs). The pathogenic potential of CNVs is supported by their larger sizes, and therefore their increased likelihood to perturb molecular mechanisms. CNVs have been implicated in the etiology of several diseases, in particular developmental disorders [11]. The effectiveness of GWAS SNPs to tag CNVs depends on the type of CNVs being assayed. Biallelic CNVs generally result from a single ancestral mutation and their tagging properties closely match those of SNPs [12]–[14]. In contrast, highly polymorphic multi-allelic CNVs may mutate frequently enough to be in low linkage disequilibrium (LD) with GWAS tag SNPs.

These observations indicate that untagged CNVs could play a significant role in the etiology of complex traits. This hypothesis is further warranted by previous reports of associations between difficult to genotype multi-allelic CNVs and HIV risk [15], as well as systemic lupus erythematosus [16]. T1D is a particularly relevant disease to assess the role of multi-allelic CNVs, owing to the established association of a variable number tandem repeat (VNTR) locus near the *INS* gene. While the *INS* locus can be used as a positive control for T1D, it is however not yet known whether the *INS* VNTR itself, or another nearby genetic variant, is actually causal [17].

Owing to our aim to assess the role of untagged CNVs, a genome-wide CNV scan needs to directly target these variants. This requirement has limited the ability to thoroughly investigate the role of CNVs for most complex traits, in particular T1D. The Wellcome Trust Case Control Consortium (WTCCC) has performed a recent large scale CNV association study using a custom designed array comparative genomic hybridization (aCGH) in a collection of 16,000 cases of eight common diseases, including T1D, and 3,000 shared controls [18]. In this effort, over 3,400 polymorphic CNVs were evaluated. While this study did not identify novel T1D associations, its coverage was limited by the relative inefficacy of the case control association to test CNVs for association. The primary reason for this limitation is that, in situations where one cannot assign discrete copy number classes to individuals at a given (‘unclusterable’) locus, subtle technical differences between cases and controls affect the CNV intensity data in a manner that inflates the false positive rate [19], [20]. Unclusterable CNVs were therefore not included in the final WTCCC CNV analysis [18], which highlights the challenge associated with assessing this class of variants. To overcome these technical limitations, we must use an alternative to the case control strategy, which motivated the use of a family design. Accordingly, novel methodologies have been developed for family based association tests that do not rely on discrete CNV genotypes but can directly incorporate raw CNV intensity data [21], [22].

To test the hypothesis that unclusterable CNVs contribute to T1D heritability, we used the collection of T1D multiplexed families recruited by the T1D Genetics Consortium (T1DGC). These samples add up to more than 4,000 transmissions between parents and affected offspring. We designed an aCGH that builds upon previous CNV discovery and genotyping experiments [12], [18], [23] but whose targets are enriched for previously untagged CNVs, for which discrete calls typically cannot be obtained. We then used this custom array to genotype the T1DGC samples and perform a genome-wide scan for T1D association.

## Results

### Array design and CNV based quality control

**Figure 1:**
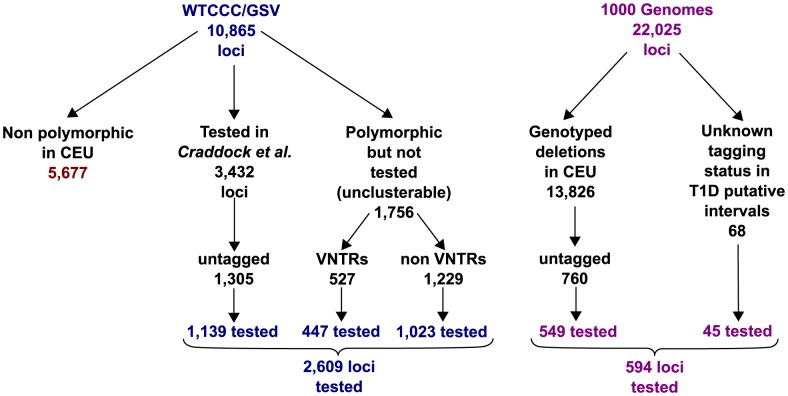
Summary of the CNVs included in the array design and tested for T1D association using FBAT-CNV. CNVs originate from two main sources: the GSV map of common CNVs [27] and the 1,000 Genomes sequence data. Tested CNVs also include 365 novel insertion CNVs obtained from the Venter genome. Detailed description of the array design is provided in Text S1.

CNVs targeted by our CGH array (Methods) were gathered from a combination of published studies of CNVs in the general population based on both CGH [12], [18] and short read genome-wide sequence data [23], [24]. Combining all sources, we targeted 4,309 CNVs (Figure 1 and Tables S1-2) that were deliberately enriched for untagged (r^2^<0.6 with best GWAS tag, Methods) and difficult to genotype loci. We were able to design probes for 4,207 loci, with the excluded loci typically being highly repeated regions in multiple genomic locations. We applied multiple QC steps to remove CNVs affected by technical artifacts (Methods). 3,410 CNV loci passed these filters. Of these, 848 CNVs were clusterable, in the sense that the intensity data could be clustered into distinct groups by the CNVtools software [19]. Table S2 lists the type of CNVs targeted by the aCGH, before and after applying QC filters. 35.9% of these 3,410 CNV loci overlap a protein coding gene (intron or exon) and 16.42% overlap a coding sequence of these genes. The median CNV size if 5.34 kb (5% quantile: 0.17 kb and 95% quantile: 64.156 kb).

### Genotyping quality and sample based quality control

Combining the four cohorts that constitute the T1DGC collection, a total of 8,460 samples were sent for genotyping (Table 1). We ensured that within each family, the same primary source of DNA (e.g. blood, cell-line) was used for every family member to minimise technical biases within families. These samples add up to 3,856 transmissions from parents to affected offspring. Following QC exclusions, 8,005 samples/3,630 transmissions were usable for the case control association tests. In addition, Illumina ImmunoChip genotype data were also available for all samples. The most common exclusion criteria were quality below the metrics suggested by Agilent QC, and non-concordance with the ImmunoChip dataset, potentially indicative of sample swaps.

**Table 1:** Number of transmissions between parents and affected T1D offspring, before/after applying QC steps.

QC step	Blood	LCL	Total	Percentage
***Before QC***	***3244***	***612***	***3856***	***100***
Agilent standard QC metrics	3075	555	3630	94.14
Consistency of familial relationships	3016	551	3567	92.51
Sample correlation	3072	555	3627	94.06
Gender check	3047	553	3600	93.36
Sample tracking	3020	555	3575	92.71
Heterozygosity	3075	555	3630	94.14
***After QC***	***3075***	***555***	***3630***	***94.14***

### Association testing strategy

**Figure 2:**
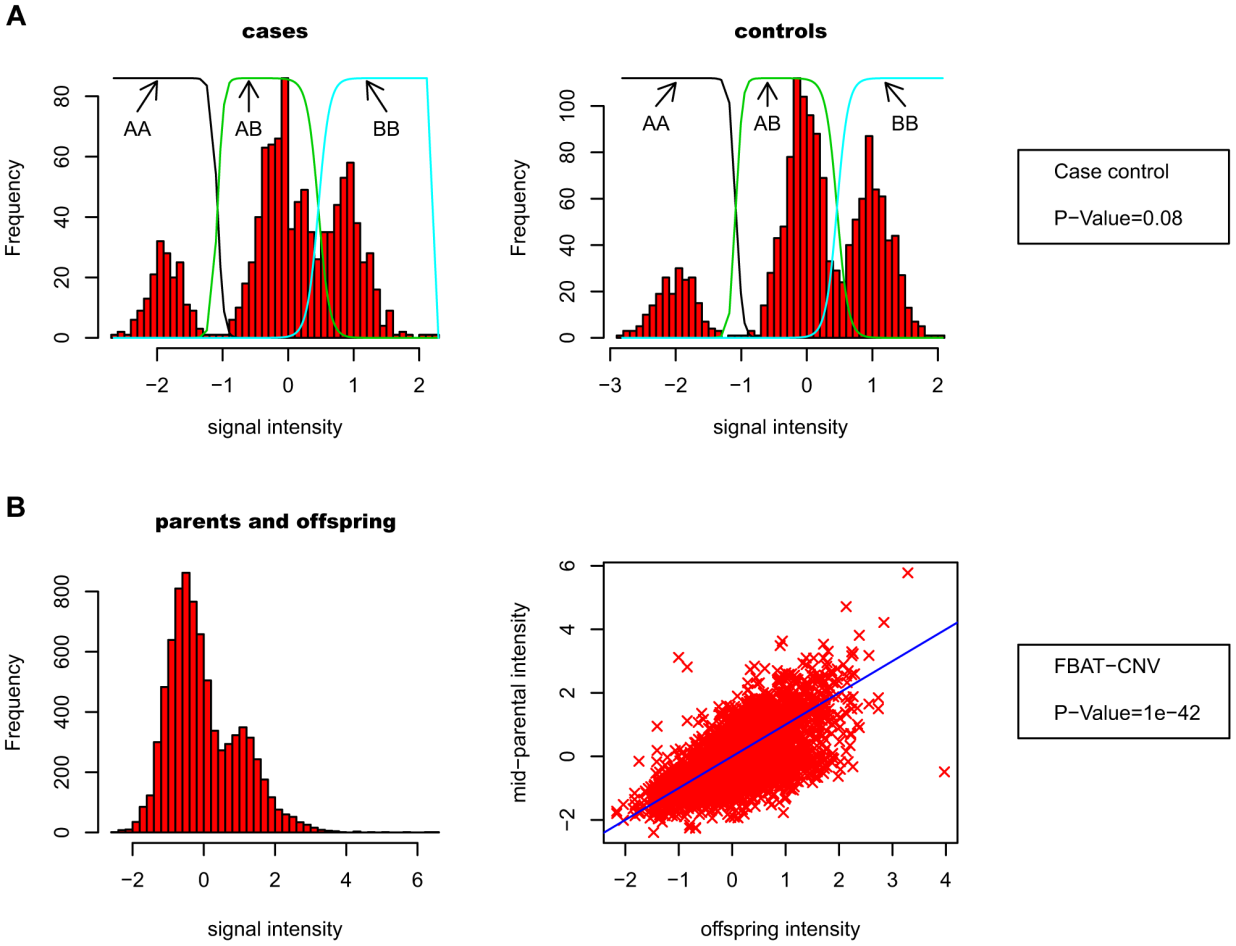
Differences between case-control and FBAT-CNV association tests. A- In a case-control analysis, technical variability may affect the CNV intensity data between cases and controls. Therefore, it is necessary to call the discrete genotypes, potentially allowing for genotype uncertainty in the association tests. Mixture models are typically used for calling, as illustrated by the colored lines on top of the histograms. Intensity data must therefore be sufficiently separated to make these discrete calls (CNV data in this example obtained from both control groups in the WTCCC study [28]). B- With the FBAT-CNV framework, one compares the average parental CNV signal with the signal for affected offspring. Consistent deviation of affected offspring intensity data compared to parental average indicates biased transmission of CNV alleles. As the test is solely based on the intensity data, and no systematic bias is expected between parents and offspring, it is not necessary to make discrete calls (CNV data obtained from INS VNTR first principal component).

Our association test strategy uses the previously developed CNV-family based association test (FBAT-CNV [22]), which is based on raw CNV intensity data rather than discrete calls. The key motivation for FBAT-CNV is to avoid the spurious false positive associations caused by technical differences between cases and controls. Instead, tests are performed within family units, comparing CNV data between affected offspring and parental average. This test does not require the CNV intensity data to be clearly separated into discrete classes (i.e. “clusterable”), in contrast with case-control association tests. Figure 2 schematically describes the differences between case-control and FBAT-CNV.

Prior to applying the FBAT-CNV test, different strategies can be used to normalize the CNV probe data, as well as summarize the intensity data from multiple probes at a single CNV locus. Overall we considered 12 different strategies, including a heritability variance scaling (HVS) scheme that builds on the probe variance scaling idea proposed in [18]. HVS maximizes the weights of probes with more heritable CNV signal (Methods). Our final pipeline selected for each CNV the optimum strategy, as measured by within family correlations (Figure S1). The rationale for this choice being that probes with heritable signals are more likely to capture true heritable CNV differences, and less likely to be affected by noise.

### Power of FBAT-CNV test

Previous investigations of the properties of the FBAT-CNV test focused on demonstrating its validity, i.e. consistency of P-value distribution under the null with theoretical expectations [21], [22]. Assessing the power of the FBAT-CNV test using theoretical arguments is likely to be assay dependent. To empirically address the issue of power, we used the subset of 22 clusterable CNVs located in the HLA- MHC chromosome region, known to be T1D associated. For these CNVs a linear standard TDT based on discrete genotypes should provide a near-optimum association testing strategy.

Figure S2 compares these TDT P-values with the result of the FBAT-CNV test. The significance of these association P-values was broadly equivalent for both testing strategies. This result suggests that, at least in the context of this study, the FBAT-CNV retains appropriate power for association testing. Unless stated otherwise, we based analyses on the FBAT-CNV association tests, independently of whether discrete genotypes were available. Table S3 lists all targeted CNVs and positions with the associated FBAT P-values.

The comparable statistical power between FBAT-CNV and TDT provides the opportunity to use established TDT power study methodology [25] to determine the range of CNVs for which our study is well powered. At a P-value threshold of P<10^−6^ a TDT with 3,610 transmissions to affected offsprings provides 80% power for bi-allelic CNVs with frequency 40% and odds ratio 1.22.

### Spurious associations at the TCR and IGH loci

**Figure 3:**
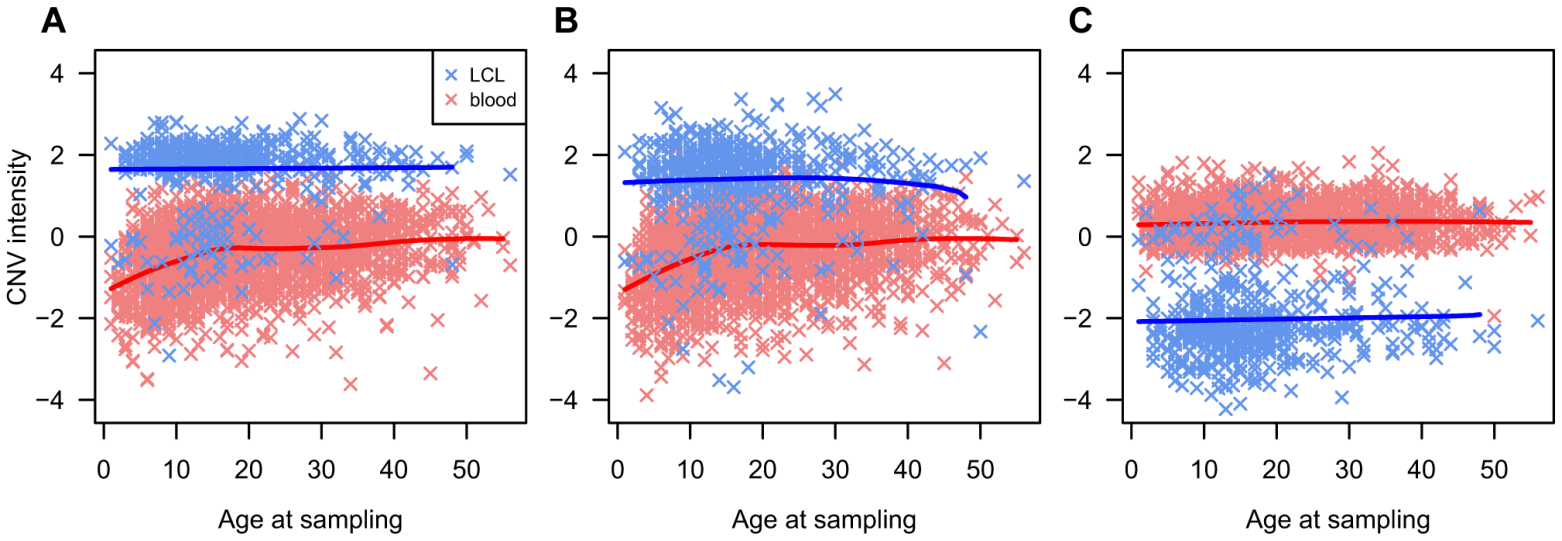
Spurious associations at TCR and IGH loci. Age at sampling (x-axis) versus CNV intensity signal (y-axis) for the three most associated Immunoglobin Heavy (IGH) and T cell receptor (TCR) loci CNVs. Each point represents an individual in the study (irrespective of familial/T1D status). Blue crosses indicate DNA extracted from LCLs (N = 551) and red crosses DNA extracted from blood (N = 2,981). Red and blue lines have been fitted to the LCL/blood data using cubic splines. A - CNVR6085.1 (chr14:21977832-21987926) mapping to TCR alpha and TCR delta locus on chr14, FBAT-CNV P = 3.6 10^−63^. The plot shows correlation between age at sampling and probe intensity for DNA extracted from blood samples. B - CNVR3590.1 (chr7:142194021-142204412) mapping to TCR beta locus on chr7, FBAT-CNV P = 4.4 10^−31^. The plot shows correlation between age at sampling and probe intensity for DNA extracted from blood samples. C - CNVR6294.22 (chr14:105433837-105441555) mapping to Ig heavy chain locus on chr14, FBAT-CNV P = 6.5 10^−5^. No age-dependent effect was detected at this locus.

We observed strong evidence of T1D association (P<10^−10^) at two loci that had been previously identified as affected by somatic rearrangements in haematopoietic lineages: CNVR6085.1, which overlaps the TCR alpha/delta (TCRA/TCRD) chain locus on chr14q11.2, and CNVR3590.1, which overlaps the TCR beta (TCRB) chain on chr7q34. Strong difference in probe intensity between DNA extracted from blood and LCLs at these loci have been previously reported [18] and our data confirm this (Figures 3A-B). However, FBAT-CNV association tests are family based and DNA source is homogeneous within the T1DGC families, hence this issue is unlikely to explain these strong associations. However, inspection of the intensity data showed correlations between age at sampling and probe intensity at both loci for DNA extracted from blood samples (Figures 3A-B). This trend is also observed in offspring after exclusion of parental intensity data. This observation suggests that the different average signal in offspring compared to parents is not a consequence of their T1D affected status, but rather of the differential age when blood was collected. We hypothesize that this trend is caused by age-dependent variability in cell type frequencies with different somatic mutation profiles. Combined with the inevitable younger age of offspring, it likely explains the strong signal of association.

In addition, moderate evidence of association (P = 6.5×10^−5^) was found at the IgG locus on chromosome 14q32.33 (CNVR6294.22, as well as several other CNVs in high LD with CNVR6294.22). We also observed at this locus a strong LCL/blood difference (Figure 3C). However, as pointed out above, tests are performed within-family and therefore this LCL-blood difference is unlikely to explain this association signal. Unlike the TCR loci, no age dependent effect was detected (Figure 3C), which leaves no explanation for this association. Nevertheless, the technical issues associated with this somatically variable locus strongly suggest that this association is the result of an unidentified technical artifact.

### Genome-wide distribution of the test statistic for T1D associations

**Figure 4:**
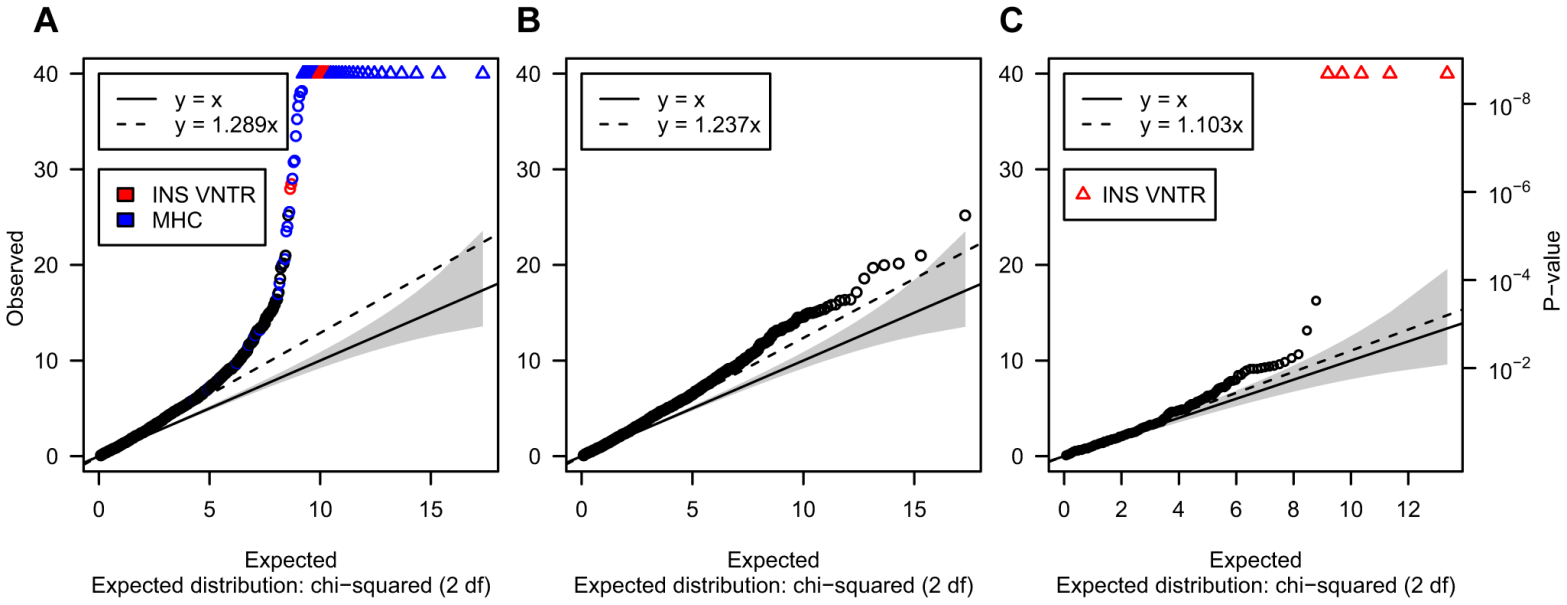
Quantile-quantile plot comparing the expected versus the observed distribution of the FBAT-CNV P-values. These plots show the distribution of -2log_10_(p), which is, under the null, distributed as chi-square with 2 degrees-of-freedom. IgG/TCR loci are discussed elsewhere and not included in these plots. A – N = 3,286 CNVs that passed quality controls and were tested for association. Loci overlapping the MHC region are marked in blue. Loci mapping to, or in strong LD with, the *INS* VNTR region are marked in red. B – N = 3,214 CNVs passed quality controls and did not overlap or tagged the *INS* VNTR and the MHC region. C – N = 448 VNTRs targeted by the CGH array that passed quality controls. *INS* VNTR CNV regions are marked in red as in Figure 3A.

Owing to our focus on CNVs difficult to genotype, and the challenge of obtaining interpretable association statistics for these loci, we initially verified that our association P-values were broadly consistent with the null hypothesis. Figure 4A shows the distribution of association statistics for 3,286 post-QC CNVs after excluding IgG/TCR loci described above. Figure 4B shows the distribution of the test statistic for the same set of loci after excluding previously reported associated CNVs (HLA-MHC, *INS* and the IgG/TCR loci). These results indicate that while some over-dispersion remains, its level is limited (over-dispersion slope of 1.23, Figure 4B). In particular, the association test statistic for 447 VNTRs that passed QC showed good concordance with the expectation under the null (over-dispersion slope 1.103, Figure 4C), with the exception of the established T1D associated *INS* VNTR, which showed unequivocal evidence of T1D association (P<10^−50^).

### Manhattan plot and genome-wide results of the scan

**Figure 5:**
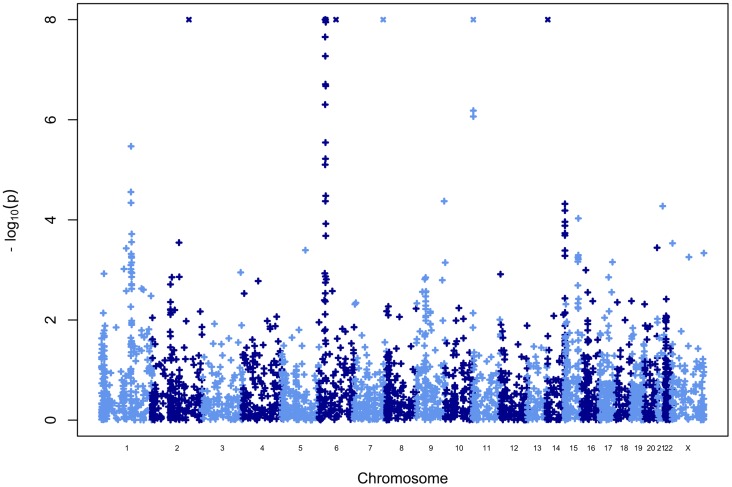
Manhattan plot for the FBAT-CNV P-values. The y-axis shows the distribution of –log_10_(p) where p is the FBAT-CNV test association test P-value for all CNV loci passing quality control filters (Methods). The x-axis shows chromosomes numbered from 1 (left) to X (right).

Figure 5 shows the Manhattan plot for the association test statistic, computed for each CNV locus. In addition to the age related effects observed at the IGH and TCR loci, two previously associated loci showed strong association results: multiple CNVs in the MHC-HLA region, as well as a smaller set of CNVs either directly targeting of located near the *INS* VNTR sequence (see below). We also identified a strong signal of association on chromosome 2q31, which has been previously identified as a duplication of this region into the HLA-MHC region, which accounts for the observed association signal.

In addition to these previously reported associations, a set of CNV loci in high LD, located in 1q21 (near the centromeric region of chromosome 1), passed the P = 0.05 Bonferroni correction threshold for 3,410 loci (corrected threshold P<1.46×10^−5^). The most associated CNV is an unclusterable duplication (CNVR341.1, Table 2, P = 3.39×10^−6^), which overlaps the first three exons of the gene *NOTCH2NL*. After excluding known loci (MHC-HLA, *INS*, *TCRA/TCRD*, *IGH*), five out of the ten most associated CNVs are located in the same 1q21 region (Table 2), hence excluding the possibility of a technical artifact that would affect a single CNV.

**Table 2:** Top ten T1D associated CNVs after removing known loci and technical artifacts.

CNV	Genomic Coordinates	Source	Type	P-value	Genes with overlapping exons
CNVR341.1	chr1:143900933-143967112	WTCCC+ unclusterable but real	duplication	3.39E-06	NOTCH2NL
CNVR335.2	chr1:143214434-143238273	WTCCC+ unclusterable but real	duplication	2.78E-05	-
CNVR4502.1	chr9:134934317-134947466	WTCCC+ untagged	duplication	4.23E-05	-
CNVR339.1	chr1:143659640-143795351	WTCCC+ unclusterable but real	duplication	4.56E-05	PDE4DIP
CNVR8001.1	chr21:35523151-35524232	WTCCC+ untagged	deletion	5.32E-05	-
CNVR6488.1	chr15:82862339-82873960	WTCCC+ unclusterable but real	deletion	9.34E-05	-
CNVR349.3	chr1:147052241-147090322	WTCCC+ unclusterable but real	duplication	1.91E-04	-
G1KSVR.1_3	chr1:146398811-146416090	G1K untagged	deletion	2.78E-04	-
CNVR986.1	chr2:130682838-130684330	WTCCC+ untagged	deletion	2.85E-04	-
CNVR8248.1	chrX:1895433-1900059	WTCCC+ unclusterable but real	duplication/deletion	2.93E-04	-

The last column lists the genes for which at least one exon overlaps the defined CNV region. P-value refers to the FBAT association test for autosomal CNVs, and to the FBAT-X association test otherwise.

### Follow-up testing for the CNVs in the 1q21 region

The suggestive evidence of T1D association in 1q21 prompted us to attempt replication for this potential finding. While the top CNV region CNVR341.1 is unclusterable, CNVR334.3 in the same genomic region is clusterable and also shows a similar strength of association (FBAT P-value 5.8×10^−4^, Table S2). Case control data from the WTCCC+ was of sufficient quality for case control association testing using the CNVtools [19] software. We found no significant association (P = 0.24) in these samples (2,000 cases, 3,000 controls, Figure S3). We hypothesized that the lack of replication could be a result of a weak effect combined with the limited power provided by the case control cohort. The previous WTCCC+ analysis indicates that rs4649771 is a good quality SNP tag for CNVR334.3. Therefore, we used a tag SNP strategy and typed this SNP (Methods) in the full JDRF/Wellcome Trust Diabetes and Inflammation Laboratory case control collection (Genetic Resource Investigating Diabetes, 7,814 cases and 9,785 controls, which included the WTCCC+ samples, Methods). We also found no evidence of T1D association (P = 0.9).

### The class I-class III alleles fully account for the INS VNTR T1D association

To understand the causal mechanism that underlies the *INS* gene/T1D association, we tiled aCGH probes across the representative sequences of all four major alleles of the VNTR in European populations (Methods). Overall, 100 aCGH tiling probes targeted the *INS* VNTR sequence. Additional genotype data for the nearby SNP rs689 (-23HphI, thought to separate class I and class III alleles) and rs3842756 (+1428 *Fok*I, thought to distinguish two subclasses of class III alleles, PH and VPH [17]) were available from the additional T1DGC ImmunoChip genotyping of the same cohort (Methods).

**Figure 6:**
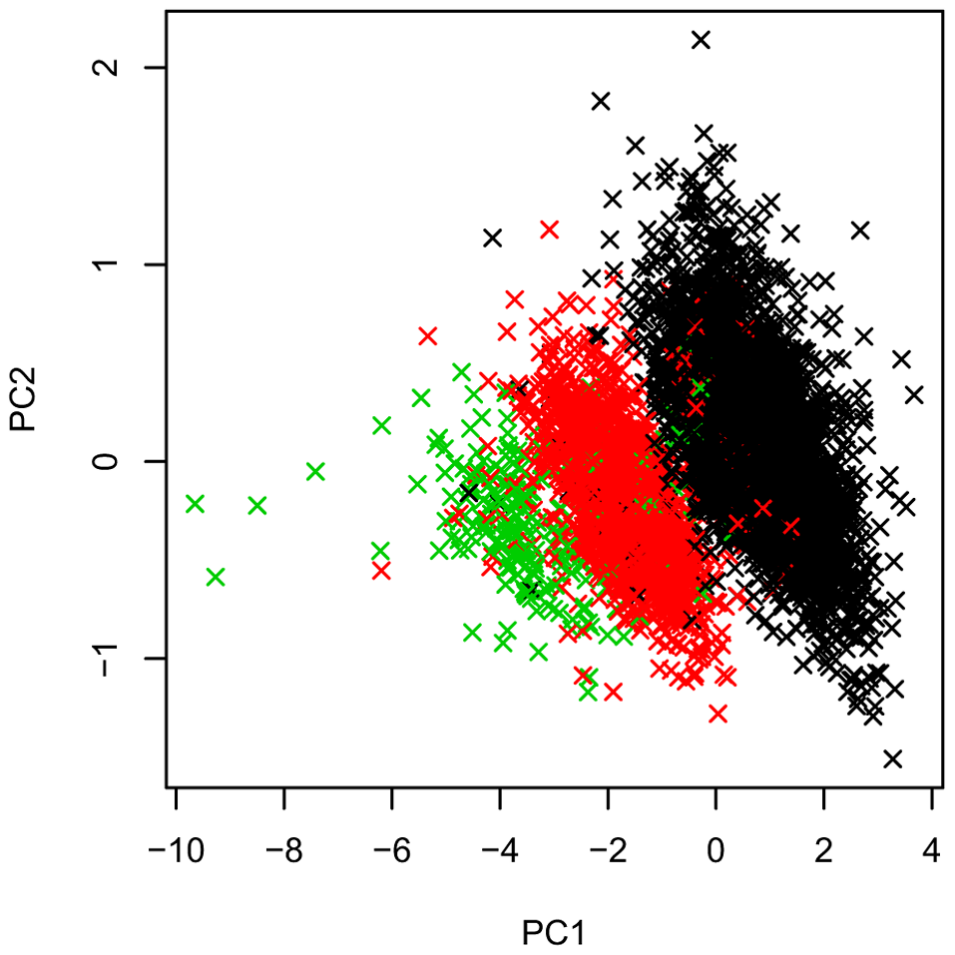
Decomposition of multi-probe CNV data at the *INS* VNTR locus into first two principal components PC1 and PC2. Principal components PC1 and PC2 summarize the multi-probe CNV data at the *INS* VNTR locus. Colors (green/red/black) were chosen based on the genotypes of the SNP rs689 (AA/AT/TT), which captures the class I-class III separation.

We decomposed the signal provided by the aCGH probes using principal component analysis (PCA). We found that PC1-PC2 captures the signal provided by the SNP rs689 (Figure 6), which is known to distinguish almost perfectly between class I and class III alleles of the VNTR [26].T1D association with rs689 was convincing (TDT test P = 5.4×10^−52^) and consistent with the FBAT CNV analysis for PC1-PC2 (P = 1×10^−42^ for PC1, and P = 1×10^−8^ for PC2)

To detect potential secondary signals at the VNTR loci from this main class I-class III effect, we performed conditional tests of association, excluding the families for which either of the parents was heterozygous at rs689. rs3842756 showed no evidence of association (P>0.05, with 1,432 transmissions from parents to affected offspring remaining). Similarly, a FBAT-CNV test for the all PCs 1-10 in that subset of individuals showed no evidence of association (P>0.05 for all, Figure S4). These results indicate that T1D association signal is driven by either rs689, or the SNP in strong LD with rs689, rs3842753 (+1140 A/C; r^2^ = 1), or the combination of PC1-PC2, both of them tagging the *INS* VNTR class I-class III alleles. Our data do not support the presence of a secondary association within the *INS* VNTR locus.

## Discussion

We performed a well-powered genome-wide scan for previously untested and untagged CNVs using a family based design. This is the first successful genome-wide association study for VNTRs and other multi-allelic CNVs. Our study targeted 3,410 CNVs, including 448 VNTRs, and a direct assessment of the role of the *INS* VNTR in T1D. The limited over-dispersion of the test statistic and the clear detection of strong association for the *INS* VNTR indicate that the family-based strategy we adopted was technically successful. These results are in stark contrast with previous genome-wide association using a case-control strategy [18], which had to exclude from the final analysis those CNVs whose raw intensity data was not clusterable into discrete genotypes. In addition to established loci and a small number of technical artifacts, a single set of CNV associations located in 1q21 passed a Bonferroni threshold of association. However, the replication results were negative, suggesting that this result is the consequence of stochastic variability rather than biologically meaningful signal. Taken together, and with the caveat that our coverage of CNVs genome-wide is not exhaustive, our results suggest that this class of CNVs does not contribute significantly to the heritability of T1D.

The *INS* VNTR is of particular interest for T1D etiology owing to its strongest effect size among all non-HLA variants, along side the *PTPN22* nsSNP rs2476601. By directly targeting this VNTR using a dense set of tiling probes, we can rule out the presence of a secondary association within the VNTR locus. The primary association is driven either by the class I-class III allele split (captured by PC1-PC2 in our PCA analysis of this locus), or by the rs689 (-23*Hph*I) variant and/or a second SNP, rs3842753, all three of them indistinguishable, as reported previously [17]. Alternative strategies will probably be required to answer the question of what the causal variant(s) and mechanism actually is, as the perfect LD in population of European ancestry will probably prevent genetic epidemiological studies in European populations from providing this answer.

While the distribution of association P-values is sufficiently close to its expectation under the null to interpret the results of our scan, the level of over-dispersion remains higher than typically reported in GWAS studies. This may partly reflect artifacts such as the ones observed at the IGH/TCR loci. In addition, for CNVs with non-linear intensity-copy number relationship, FBAT-CNV, departure from Hardy-Weinberg equilibrium (HWE) can inflate the type 1 error [21]. Hence, population structure in the parental samples leading to departure from HWE could explain the moderately biased distribution of the FBAT-CNV association statistics.

Here, we have extended the set of genetic variants considered in T1D association studies to include a major novel category of CNVs. However, this set is still limited by the requirement to design short oligonucleotide probes for the aCGH assay that have minimal matches elsewhere in the genome. Shorter repeats regions, in particular microsatellites, remain out of reach of genome-wide association tests using array-based technologies. The typically higher mutation rate of microsatellites could lead to a mutation-selection equilibrium that is compatible with larger odds ratio of association for T1D. Targeting these variants will require large scale sequencing studies with sufficiently long reads to characterize these difficult loci reliably. Future improvements of sequencing technologies will provide an opportunity to further broaden the scope of association tests in the future.

## Materials and Methods

### CNV selection

CNVs selected as targets originated from the following sources: (i) Loci from the WTCCC+ genotyping chip that were classified as successfully genotyped but at the same time untagged by SNPs in the WTCCC+ study, (ii) Loci from the GSV/WTCCC+ set that could not be genotyped but that were classified as real polymorphisms in the WTCCC+ study, (iii) Loci from the 1000 Genomes project union set of deletions from Pilot 1 (low coverage samples) and Pilot 2 (high coverage trio samples) phases that were genotyped but were not tagged by SNPs in the 1000 genomes project (PhaseI + PhaseII), (iv) Loci from the 1000 Genomes project union set of deletions from Pilot 1 (low coverage samples) and Pilot 2 (high coverage trio samples) phases of the 1000 genomes project for which the SNP tagging status was unknown and lied in T1D association intervals, (v) Insertions of novel sequences present in the genome sequence of Craig Venter but not in the reference sequence that were not previously tested for association in the WTCCC+ study, (vi) candidate gene loci PRDM9 VNTR and NCF1 gene, (vii) functional elements in T1D intervals, (viii) Control loci from WTCCC1 X-chromosome CNV desert regions and from a set of CNV loci genotyped in the WTCCC+ study or in the 1000 Genomes project to facilitate detection of sample mishandling. A more detailed breakdown of the target loci is given in Text S1 and Figures S5-7. Table S4 provides the bed file that was sent to Agilent for array design.

### Samples

The Type 1 Diabetes Genetics Consortium (T1DGC) represented an international effort to identify genes that determine an individual's risk of T1D. A major effort of the T1DGC was the creation of a resource base of well-characterized families from multiple ethnic groups to facilitate the localization and characterization of T1D susceptibility genes. After consideration of previous genome-wide linkage scan results for T1D, the contributions of known T1D risk loci to familial clustering of T1D, and the power of affected sib-pair linkage studies, the T1DGC proposed to assemble a collection of 4,000 affected sib-pair (ASP) families, requiring de novo recruitment of ∼2,800 ASP families. In order to identify, recruit, and collect the samples and data on these newly ascertained ASP families, the T1DGC developed four regional networks: North America (NA), Europe (EU), United Kingdom (UK), and Asia Pacific (AP). Within each network, field centers identified, ascertained, and collected samples and data from participating families, with samples shipped and processed in laboratories at multiple sites. Each network had a repository to process samples for DNA and to provide cell immortalization using standard protocols. Samples and data from the T1DGC network laboratories have been transferred to the central NIDDK repository (www.niddkrepository.gov) from which investigators can make requests.

Protocols and informed consents for sharing of data and samples were approved by review boards of all contributing institutions, and appropriate informed consent was obtained from families. Briefly, a family must contain at least one affected sib-pair; “affected” indicates a diagnosis of T1D in the proband (index case) before the age of 35 years, with insulin required within 6 months of diagnosis. Two sources of DNA were available, blood (DNA was extracted from cell pellet collected during plasma isolation from blood) and EBV-transformed lymphoblastoid cell lines (LCLs). Only families with one source of DNA were included (all members with blood DNA (N = 7,152) prioritized over all members with cell line DNA (N = 1,308)). From this filtering, 8,460 individuals were included for the CNV study, 1,661 families with at least four members (2 parents and 2 affected children, n = 6,963) and 499 families with three members (2 parents and an affected child, n = 1,497).

### DNA processing

The T1DGC DNA stock plates were thawed to room temperature, typically for at least three hours to ensure that the DNA was homogeneous. The stock plates were vortexed at 1000 rpm for 1 minute, then centrifuged at 280 xg for 1 minute. Stock DNA concentrations were determined using a Nanodrop spectrophotometer. An 18 ul sample from the DNA stock plate was transferred to a CNV dilution plate, after which all samples were normalized using 10 mM Tris either at University of Virginia (Charlottesville, VA) or the OGT facility (Oxford, UK). Once the samples were diluted, the plates were vortexed at 1000 rpm for 1 minute and centrifuged at 280 xg for 1 minute. The plates were allowed to incubate at room temperature for 30 minutes. DNA concentration of the CNV dilution plates were evaluated using a Nanodrop spectrophotometer, targeting a range of 60–70 ng/ul (acceptable was considered 40–80 ng/ul). For samples out of range (e.g., a sample higher than 80 ng/ul), more Tris buffer was added, mixed, and reassessed. Once all of the samples were within the desired concentration range, the CNV dilution plates were sealed and stored them at 4°C. A Biomek FX robot was used to transfer 15 ul of each sample from the CNV dilution plates to daughter plates, that were then heat-sealed and stored at −20°C until shipped to Oxford Gene Technology (OGT, Oxford, England) and processed.

### aCGH data generation

Data were generated using the Agilent 8×60k custom array CGH at the OGT facility (Oxford, UK). Samples were platted in rows. Families were processed within the sample plates, with family members in consecutive wells and random permutations to change the position of the family members on the columns. All family members were processed on the same custom array slide. The shared reference DNA sample for the entire study is a pool of genomic LCL DNA from 10 male HapMap subjects (NA06994, NA07051, NA11992, NA12003, NA12043, NA12045, NA12144, NA12155, NA12750, NA12891). Equal amounts of test and reference DNA were used in the DNA labeling reactions and hybridized to the custom array. Following hybridization the arrays were scanned using the red channel for the test sample and the green channel for the reference sample. An internal control reaction was carried out on each plate to assess DNA labeling and hybridization steps.

### Data processing

Prior to data analysis we normalised the data in 6 different ways by applying some transformations of the red (R) and green (G) raw copy number intensities as proposed in [18] (QNorm refers to quantile normalized intensity data):

1. log(R/G)
2. log(QNorm(R)/QNorm(G))
3. QNorm(log(R/G))
4. QNorm(log(QNorm(R)/QNorm(G)))
5. log(R/G+0.5)
6. log(QNorm(R)/QNorm(G) + 0.5)

Multiple probes at a single CNV were summarized using a PCA analysis (without rescaling the probe data to unit variance). In addition, it has been pointed out before [18] that the contribution of each probe to the intensity signal is variable. We took advantage of the family design to increase weight for probes likely to be more informative, a method we called heritability probe scaling (HPS). Precisely, for each probe on the array, we estimated correlation between offspring and mid-parental intensities. HPS then rescales probe intensities proportionally to this measure of heritability.

Combining these 6 normalizations and 2 different probe summary techniques (PCA and HPS-PCA), we considered 12 different data summaries per CNV. At each of the 4,201 CNV loci, we selected the method among these 12 that maximized the correlations between mid-parental intensity and used this choice in the association tests (Figure S1).

### Case-control dataset of replication

Case subjects were diagnosed with T1D before 17 years of age (mean age at diagnosis 7.8 years) and were from the Juvenile Diabetes Research Foundation/Wellcome Trust Diabetes and Inflammation Laboratory Genetic Resource Investigating Diabetes study (www.childhood-diabetes.org.uk/grid.shtml). Control subjects were obtained from the British 1958 Birth Cohort (www.cls.ioe.ac.uk/studies.asp?section=000100020003) and the Wellcome Trust Case-Control Consortium U.K. Blood Service Common Control sample collection. All samples were of white European ancestry.

### Genotyping replication of rs4649771

rs4649771 was genotyped using TaqMan nuclease assay (Applied Biosystems, Warrington, U.K.) according to the manufacturer's protocol. Genotyping was performed blind to case-control status and double scored to minimize error. Primers and probes were as follows: forward primer GCAGTTTAGGGTCTAATGAGGTAAGTG, reverse primer CCCTGGGTATCTAGGTACCTTATCA, FAM probe CAGCATCCAATAGAAGT, VIC probe CAGCATCCAACAGAAGT.

### Quality controls

#### Agilent metrics

An initial set of Agilent quality metrics for red and green raw intensity probe signals was used to flag problematic samples. Signal intensity, background noise, signal to noise ratio and derivative log ratio spread (DLRS, which measures the spread of log ratio difference between consecutive probes) summarised data quality across all probes and provided us with an initial quality assessment independent from subsequent processing step. Metrics were ranked as ‘Excellent’, ‘Good’ or ‘Poor’ based on the thresholds proposed by OGT. Samples with at least one metric flagged as ‘Poor’ were excluded from further analysis.

#### Sample correlation

To identify potential sample swaps, we computed Pearson's correlation coefficient between pairs of samples using n = 452 clusterable CNVs. Based on the overall distribution of correlation coefficients (Figure S8) we selected a cut-off of 0.7 to flag pairs of samples as correlated. Samples showing correlation greater than this cut-off were flagged as problematic.

#### Consistency of familial relationships

To identify inconsistent maternal and paternal relationships, we used a likelihood-based approach. We fitted a model H_0_ of true paternity (resp. maternity) and a model of non-paternity H_1_ (resp. maternity) to the genotype data from N = 318 clustered CNVs with MAF >0.3. We used a Bayes factors threshold of 100 in favor of non-paternity to flag likely problematic parent-offspring relationships.

#### Heterozygosity

We used genome-wide sample heterozygosity as a criteria to exclude samples from the analysis. We computed the heterozygosity based on a set of 310 autosomal clusterable CNVs with high confidence genotype calls (posterior probability >95% based on CNVtools output). We plotted the average number of heterozygous against the genotype call rate. Samples with heterozygosity z-score <−5 and call rate <98% or heterozygosity z-score >2 and call rate <92% were flagged as outliers for the analysis (Figure S9).

#### Gender check

We used a set of 10 regions without annotated CNVs on chromosome X to determine the number of copies of chromosome X and therefore infer gender. This QC step together with sample tracking led to the identification of a full plate swap between plate 116 and 118 which could be recovered.

#### Sample tracking

Sample tracking was performed on a set of 40 CNVs genotyped by either the WTCCC+ or the 1000 Genomes project with CEU MAF >10% that are well-tagged (r^2^>0.9) by SNPs present on the ImmunoChip (Figure S10). A threshold of 80% concordance was used to flag outliers.

### QC strategy

A merged list of flagged samples was generated, and whenever possible manual inspection was used to resolve problematic samples. Any sample that failed at least one of these quality tests and that could not be resolved was discarded from the analysis. We also checked that plate rows and columns showed comparable CNV intensity data and did not find any systematic change (Figure S11).

### Association testing method

Association tests use the FBAT strategy [21], [22] implemented in the version 2.0 of the CNVtools [19] package. For chromosome X CNVs not located in the pseudo-autosomal region, we only compared the CNV intensity between daughters and mothers (hence restricting the data to approximately half of the sample size).

## Supporting Information

Figure S1 Heritability as a pipeline selection strategy. A measure of heritability is used to select the optimal pipeline for each CNV locus. Each boxplot quantifies heritability for a given pipeline (from N1 standing for normalised1 to N6 standing for normalised6, see Methods for a description of these normalization choices). Boxplots are ordered according to increasing heritability. Pipelines whose data is rescaled according to its first principal component are marked in blue, whereas pipelines whose data is rescaled according to heritability probe scaling are marked in dark green. The magenta horizontal line represents the average heritability measured across all pipelines. The optimal pipeline selects the most heritable pipeline at every CNV locus. The rationale for this choice is that probes with heritable signals are more likely to capture true heritable CNV differences, and less likely to be affected by noise.
(EPS)

Figure S2 Comparison of association P-values using FBAT-CNV and discrete genotyping calls for T1D associated CNVs located in the MHC complex. In order to empirically assess the power of family based association tests a subset of clusterable CNVs known to be positively associated with T1D was used to compare intensity-based FBAT-CNV P-values with genotype-based TDT P-values. **A** - Comparison of association P-values using FBAT-CNV (x-axis) and TDT (y-axis) for N = 22 T1D associated CNVs located in the MHC complex. **B** - Zoom in on the left-bottom corner of Figure S2A.
(EPS)

Figure S3 Family and case control (WTCCC+) replication data for CNVR341.1 and for its best clusterable tagging CNV CNVR334.3. **A** - Top association in the T1D familial cohort for unclusterable CNVR341.1 (FBAT-CNV P-value = 3.39 10^−6^). **B** - Intensity data for CNVR341.1 in the WTCCC+ case control data (unclusterable, hence no association P-value available). **C** - Intensity data for the clusterable CNVR334.3 (FBAT-CNV P = 5.8 10^−4^) in the same 1q21 region in the T1D familial cohort. **D** - Intensity data for the correlated CNVR334.3 (CNVtools case-control P = 0.24) in the WTCCC+ case control dataset.
(EPS)

Figure S4 Primary and conditional on rs689 FBAT-CNV test of association for *INS* VNTR. **A** - First principal component PC1 intensity data for *INS* VNTR (FBAT-CNV P-value = 10^−42^. **B** - Third principal component PC3 intensity data for *INS* VNTR after excluding families where either of the parents is heterozygous at SNP rs689 (FBAT-CNV P-value = 0.82).
(EPS)

Figure S5 Mixed probe design with breakpoint and internal sequence probes. One of the innovations of the CGH array is the introduction of a mixed probe design consisting of both quantitative internal sequence probes and qualitative breakpoint probes for loci with known breakpoints, as opposed to standard CGH probe design where all probes are internal to the CNV locus.
(EPS)

Figure S6 Design of probes over CNV breakpoints. Each locus with known breakpoint is targeted by three qualitative probes: one probe spanning the breakpoint junction on the alternate non-reference allele (ALT) and two probes spanning each one of the breakpoints on the reference allele (REF). To maximize discrimination between the three sequence probes, these probes are designed to be centered on different locations that depend on whether the targeting breakpoint is classified as blunt, micro-homology (µH) or non-templated sequence (NTS). If the breakpoint is blunt the ALT probe is centered on the breakpoint, whereas the left-hand reference probe and the right-hand reference probe are respectively centered on the start and on the end of the breakpoint on the REF sequence. If the breakpoint is a micro-homology, the ALT probe is centered on the micro-homology, the left hand-side REF probe is centered on the end of the micro-homology and the right hand-side REF probe is centered at the end of the breakpoint. If the breakpoint is categorized as non-template sequence, the ALT probe is centered on the midpoint of the non-templated sequence, whereas the left-hand REF probe and the right-hand REF probe are respectively centered on the start and on the end of the breakpoint on the reference sequence.
(EPS)

Figure S7 Breakpoint probe design examples. Examples of reference (red) and alternate (green) alleles and breakpoint probe positioning for respectively blunt, micro-homology and non-templated sequence deletions. Start and end refer to start and end of breakpoint detected in reference allele. In case of blunt deletions the left-hand (right-hand) side reference probe will bind to the start (end) of the deletion on the reference allele, and the alternate probe will bind to the deletion breakpoint on the alternate allele. In case of micro-homology deletions, under the design assumption that the second micro-homology is being deleted, the left-hand side reference probe will bind to the end of the first micro-homology on the reference allele, the right-hand side reference probe will bind to the end of the deletion on the reference allele and the alternate reference probe will bind to the middle of the micro-homology on the alternate allele to maximize discrimination between breakpoint probe sequences. In case of non-template sequence deletions, where a sequence is inserted at the breakpoint, the left-hand (right-hand) side reference probe will bind to the start (end) of the deletion on the reference allele, and the alternate probe will bind to the middle of the non-template sequence on the alternate allele to maximize discrimination between probe sequences.
(EPS)

Figure S8 Distribution of R^2^ correlation between pairs of unrelated individuals. Squared Pearson correlation coefficient (R^2^>0) is measured between pairs of unrelated samples across 452 clusterable CNVs. A cutoff of R^2^ = 0.7 is used to flag highly correlated samples likely to result from technical artifacts (such as aliquoting issues).
(EPS)

Figure S9 Genotype call rate versus heterozygosity. Heterozygosity z-score (x-axis) is plotted against genotype call rate (y-axis) based on a set of 310 autosomal clusterable CNVs with high confidence genotype calls (95% posterior probability based on CNVtools output). Each point is a sample. Red samples (z-score <−5 and genotype call rate <98% or z-score >2 and genotype call rate <92%) are flagged as problematic.
(EPS)

Figure S10 Genotype concordance between CNV genotypes and ImmunoChip genotypes. A set of 40 CNVs genotyped by either the WTCCC+ or the 1000 Genomes project with CEU MAF>10% that are well-tagged (R^2^>0.9) by SNPs present on the ImmunoChip is used for sample tracking. Each datapoint in the plots is a sample. The x-axis represents plate position and the y-axis represents genotype concordance between genotypes based on CNVtools posterior probabilities and ImmunoChip genotypes. The scatterplot on the left-hand side (**A**) show the distribution of genotype concordance under the null hypothesis of no concordance with ImmunoChip genotypes. The right-hand side scatterplot (**B**) shows the estimated genotype concordance with ImmunoChip genotypes. A threshold of 80% concordance is used to flag outliers. The long vertical line at the bottom of the right-hand side plot flags two full plate swaps.
(EPS)

Figure S11 Distribution of raw un-normalized red intensity signal across plates for samples passing quality controls. Plate rows and columns show comparable CNV intensity data before data pre-processing and normalization. **A** - Boxplots of raw red intensity signal across CGH array plate rows E,F,G and H. **B** - Boxplots of raw red intensity signal across CGH array plate columns 1 to 12.
(EPS)

Table S1 Source of CNVs targeted by the aCGH, before and after applying QC filters.
(CSV)

Table S2 Type of CNVs targeted by the aCGH, before and after applying QC filters.
(CSV)

Table S3 Targeted CNVs with associated FBAT P-values.
(CSV)

Table S4 BED file for T1D CNV array design.
(TXT)

Text S1 Description of array and probe design.
(DOCX)
