# Supplementary material for: A Genome-Wide Assessment of the Role of Untagged Copy Number Variants in Type 1 Diabetes
Source: PLoS Genet. 2014 May 29;10(5):e1004367. doi: 10.1371/journal.pgen.1004367 (PMC4038470; doi:10.1371/journal.pgen.1004367)
Supplement: Text S1 — Description of array and probe design. (DOCX) [file pgen.1004367.s016.docx]

**Supporting Online Material, Text S1: T1D CNV Array design**

*CNV selection*

CNVs selected as targets originated from the following sources: (i) Loci from the WTCCC+ genotyping chip that were classified as successfully genotyped but at the same time untagged by SNPs in the WTCCC+ study, (ii) Loci from the GSV/WTCCC+ set that could not be genotyped but that were classified as real polymorphisms in the WTCCC+ study, (iii) Loci from the 1000 Genomes project union set of deletions from Pilot 1 (low coverage samples) and Pilot 2 (high coverage trio samples) phases that were genotyped but were not tagged by SNPs in the 1000 genomes project (PhaseI + PhaseII), (iv) Loci from the 1000 Genomes project union set of deletions from Pilot 1 (low coverage samples) and Pilot 2 (high coverage trio samples) phases of the 1000 genomes project for which the SNP tagging status was unknown and lied in T1D association intervals, (v) Insertions of novel sequences present in the genome sequence of Craig Venter but not in the reference sequence that were not previously tested for association in the WTCCC+ study, (vi) candidate gene loci *PRDM9* VNTR and *NCF1* gene, (vii) functional elements in T1D intervals, (viii) Control loci from WTCCC1 X-chromosome CNV desert regions and from a set of CNV loci genotyped in the WTCCC+ study or in the 1000 Genomes project to facilitate detection of sample mishandling.

*(i) Untagged CNVs in the WTCCC+ study*

A CNV locus from the WTCCC+ study was defined to be untagged if a maximum squared Pearson’s correlation coefficient r^2^ tag could be calculated for both Illumina 660W and Affy 550K platform data and the sum of these tags was less than 1.2. WTCCC+ and 1000 Genomes loci were scanned for reciprocal overlap. Ultimately a set of 1,305 WTCCC+ genotyped but untagged CNVs were included in the array design, 265 of which could be associated with breakpoints from the 1000 Genomes deletions.

*(ii) Untyped CNVs in the WTCCC+ study*

A CNV locus from the WTCCC+ genotyping chip was defined as untyped but real in the WTCCC+ study if it satisfied all the following criteria: (i) it could be validated by the GSV study [1], (ii) it could be discovered in at least 1 CEU sample in the GSV study, (iii) it could not be clustered in the WTCCC+ study, (iv) it was non redundant, i.e. it did not share all its mapping probes with another CNV locus from the WTCCC+ study, (v) it had a MAD ratio [2] from the WTCCC+ study lower than 0.75. According to these criteria, a set of 1,651 WTCCC+ loci was identified, of which 48 could be associated with breakpoints from the 1000 Genomes study, and of which 422 were VNTRs. 105 additional annotated VNTRs that met all these criteria except the MAD ratio threshold were also added to the list of target CNVs.

*(iii) Untagged CNVs in the 1000 Genomes dataset*

This is a subset of the 13,826 genotyped deletions from the union of Pilot 1 (low coverage samples) and Pilot 2 (high coverage trio samples) phases of the 1000 Genomes project. A deletion from the union of deletions from Pilot 1 and 2 from the 1000 Genomes project was defined as untagged if no SNP with r^2^ genotype correlation greater or equal than 0.6 could be identified for this locus in the following manner. Only deletions that were typed in at least 1 out of 44 CEU unrelated samples. After merging overlapping CNV regions, a set of 760 genotyped but untagged 1000 Genomes deletions were selected for inclusion in the array design, of which 575 had annotated breakpoints.

*(iv) 1000 Genomes CNVs with unknown tagging status in T1D intervals*

This is a subset of the 22,025 merged deletions from the union of deletions discovered in pilot 1 (low coverage samples) and pilot 2 (high coverage trio samples) phases of the 1000 Genomes project. A deletion from this subset was selected for inclusion if (i) it was discovered in at least 1 CEU sample from the1000 Genomes Project, (ii) it had unknown tagging status (i.e Pearson correlation coefficient could not be assayed), (iii) it showed at least 50% reciprocal overlap with one of the T1D association intervals identified in previous T1D studies. Two deletions showed more than 50% reciprocal overlaps with ALU and LINE elements and were removed. Seven large deletions (>50 kb) were already captured by our coverage of T1D regions (see below) and therefore not included in this additional list. Together, this category identified 68 additional deletions, of which 21 had annotated breakpoints.

*(v) Novel sequence insertions.*

We sought to identify copy number variable sequences not present in the reference sequence (NCBI36) by investigating novel sequences that had been previously identified in in the Craig Venter genome [3]. Starting from a set of 471 insertions of novel sequences having less than 40% of repeat-masked sequence and at least 1Kb of non-repeat-masked sequence we filtered out loci that had already been already been assayed in the WTCCC+ project. We eventually selected a set of 365 novel sequences, selected on the basis of longer length of non-repeat masked sequence.

*(vi) Candidate gene loci*

We sought to investigate *NCF1* and *PRDM9* VNTR, two candidate genes located in complex regions of the human genome that were not previously tagged by SNPs. *NCF1* lies in a larger region of segmental duplication which has been implicated in rheumatoid arthritis [4] and for which there is some evidence of recent duplication for *NCF1* in the human lineage [5]. We selected a total of three loci targeting the segmental duplication region upstream of the gene, the actual gene region, and the segmental duplication region downstream of the gene. *PRDM9* VNTR was selected under the basis that its variation has been shown to influence recombination hotspot activity in humans [6].

*(vii) Functional elements in T1D intervals*

A secondary aim of this array was to probe known T1D regions for CNVs. We identified 8,934 putative functional elements that lie within known T1D associated intervals [7]. T1D regions were obtained from T1D base version 4.2 ([www.t1dbase.org](http://www.t1dbase.org)), where putative regions are constructed starting from published T1D variants that are repeatedly extended out by ±0.1cM and further investigated for variants of genome-wide significance until no more variants reach a significance of 1E-06 within cited study. If part of a gene lied in a T1D association interval then all exons of the gene were included, irrespective of whether the exon itself lied within the association interval. These functional elements were defined using both conservation (Genome Evolutionary Rate Profiling – GERP score, version 31) and gene annotation criteria obtained from Ensembl54; overlapping elements were then merged to generate a non-redundant set. Some of these loci were further extended by 100 base pairs upstream and downstream to merge with nearby target regions. After merging, 7,777 extended functional elements were selected for inclusion in the array design.

*(viii) Control loci*

A set of 50 CNVs were included in the CGH array for the sole purpose of facilitating the automated detection of sample mishandling. A set of 10 X-chromosome CNV desert regions from the WTCCC+ study was included to determine chromosome X dosage from T1DGC intensity data. A set of 40 CNVs that are well typed in the WTCCC+ typing array or the 1000 Genomes project with a MAF> 10% in the CEU population and that are also well-tagged r^2^>0.9) by SNPs from the ImmunoChip genotyping array was included to allow for sample tracking.

***Removing redundancy from targeted loci***

A preliminary analysis of candidate loci identified some level of redundancy (reciprocal overlap) between 1000 Genomes deletions occurring in regions of large segmental duplication as well as between 1000 Genomes deletions and WTCCC+ loci. To maximize the number of unique genomic regions that could be targeted by the aCGH, we refined the initial set of loci to retain only the union of most unique regions between WTCCC+ loci and 1000 Genomes project in the following manner. Clusters of highly correlated (r^2^>0.8) 1000 Genomes deletions were merged and represented by the largest most redundant interval that could be identified within the cluster. Following this step we proceeded to filter out from the set of candidate loci any WTCCC+ locus showing at least 50% reciprocal overlap with 1000 Genomes.

***Mixed design with breakpoint and internal sequence probes***

A methodological innovation used in this study is a mixed probe design

that combines traditional probes (targeted to the copy number variable sequence, which we refer to as internal probes) as well as breakpoint probes in cases where these breakpoints are known (Figures S5, S6 and S7).

Internal probes were selected on basis of Agilent in silico probe performance, which has been previously shown to correlate with empirical performance [2]. Breakpoint junction probes were designed by providing Agilent with three informative sequences per deletion with known breakpoints: one for each side of the undeleted allele and one for the junction of the deleted allele.

***Assigning 1000 Genomes breakpoints to WTCCC+/GSV loci***

To maximize the number of loci with junction breakpoint probes we inferred WTCCC+ CNV breakpoints by looking at their reciprocal overlap with 1000 Genomes deletions that were not included in the aCGH. To assign such putative 1000 Genomes breakpoints to WTCCC+ CNVs we required at least 50% reciprocal overlap with a 1000 Genomes deletion. If a WTCCC+ CNV overlapped multiple 1000 Genomes deletions with known breakpoints, we assigned the breakpoints of the deletion with the highest reciprocal overlap.

***Probe design***

To maximize the number of targeting loci, we reduced the number of Agilent standard control probes from 3,886 to 853, by keeping only probes strictly required by the Agilent feature extraction software to work. For each target CNV without known breakpoints (this excludes functional elements in T1D intervals and quality control regions), 10 different internal probes were sought. If only 5 good quality probes could be designed, then the best scoring probes would be replicated to make up to ten. Otherwise, the locus was not included in the T1D CNV array. If a CNV had known breakpoints, only 7 internal probes were sought, with the requirement that at least 3 unique good quality probes could be designed and that the top quality probes could be replicated to make up to 7, otherwise the locus would be discarded. In addition to these 7 probes, 3 breakpoint probes were designed from 121 base-pair template sequences centered around the central base of each of the three breakpoint informative sequences. The central base of each sequence was chosen according to the sequence context of the deletion breakpoint (blunt, micro-homology or non-template sequence) as shown in Figure S6 and S7. Following these design requirements it was possible to design probes for 97.6% of targeted CNVs (i.e. 4,207 of the 4,309 CNVs, Table S1). The targeted regions provided to Agilent for probe design are given in BED file format in Table S4, where genomic coordinates (human assembly hg18 – NCBI 36.1) are listed for all designed loci, all loci that could be ultimately targeted in the array and their relative mapping probes.

***Probe design for INS VNTR***

For the *INS* VNTR locus, representative allelic sequences from the 4 major alleles in UK individuals (IC, ID, IIIA, IIIB) were reconstructed from repeat sequences and allele repeat codes given in [8], and checked against MVR-PCR codes for these allele classes from [9]. Tiling probes were sought every 28 base-pairs across 4 representative alleles from each major subclass of VNTR alleles for the UK population.

**References**

1. Conrad DF, Pinto D, Redon R, Feuk L, Gokcumen O, et al. (2010) Origins and functional impact of copy number variation in the human genome. Nature 464: 704–712. Available: http://dx.doi.org/10.1038/nature08516. Accessed 18 July 2011.

2. Craddock N, Hurles ME, Cardin N, Pearson RD, Plagnol V, et al. (2010) Genome-wide association study of CNVs in 16,000 cases of eight common diseases and 3,000 shared controls. Nature 464: 713–720. Available: http://www.pubmedcentral.nih.gov/articlerender.fcgi?artid=2892339&tool=pmcentrez&rendertype=abstract. Accessed 11 June 2011.

3. Levy D, Ehret GBB, Rice K, Verwoert GCC, Launer LJJ, et al. (2009) Genome-wide association study of blood pressure and hypertension. Nat Genet. Available: http://dx.doi.org/10.1038/ng.384.

4. Olsson LM, Holmdahl R (2012) Copy number variation in autoimmunity--importance hidden in complexity? Eur J Immunol 42: 1969–1976. Available: http://www.ncbi.nlm.nih.gov/pubmed/22865047. Accessed 18 December 2013.

5. Sudmant PH, Kitzman JO, Antonacci F, Alkan C, Malig M, et al. (2010) Diversity of human copy number variation and multicopy genes. Science 330: 641–646. Available: http://www.pubmedcentral.nih.gov/articlerender.fcgi?artid=3020103&tool=pmcentrez&rendertype=abstract. Accessed 11 December 2013.

6. Berg IL, Neumann R, Lam K-WG, Sarbajna S, Odenthal-Hesse L, et al. (2010) PRDM9 variation strongly influences recombination hot-spot activity and meiotic instability in humans. Nat Genet 42: 859–863. Available: http://www.pubmedcentral.nih.gov/articlerender.fcgi?artid=3092422&tool=pmcentrez&rendertype=abstract. Accessed 13 December 2013.

7. Barrett JC, Clayton DG, Concannon P, Akolkar B, Cooper JD, et al. (2009) Genome-wide association study and meta-analysis find that over 40 loci affect risk of type 1 diabetes. Nat Genet 41: 703–707. Available: http://dx.doi.org/10.1038/ng.381.

8. Bennett ST, Todd JA (1996) Human type 1 diabetes and the insulin gene: principles of mapping polygenes. Annu Rev Genet 30: 343–370. Available: http://www.ncbi.nlm.nih.gov/pubmed/8982458. Accessed 9 September 2013.

9. Stead JD, Buard J, Todd JA, Jeffreys AJ (2000) Influence of allele lineage on the role of the insulin minisatellite in susceptibility to type 1 diabetes. Hum Mol Genet 9: 2929–2935. Available: http://www.ncbi.nlm.nih.gov/pubmed/11115836. Accessed 9 September 2013.
